# Navigating the Lay Referral System for Treatment‐Seeking Decisions During Illness in the Digital Age: A Qualitative Study of Adults Living in Slums in Nigeria

**DOI:** 10.1111/1467-9566.13863

**Published:** 2024-11-30

**Authors:** Chinwe Onuegbu, Jenny Harlock, Frances Griffiths

**Affiliations:** ^1^ Division of Health Sciences Warwick Medical School University of Warwick Coventry UK; ^2^ Centre for Health Policy University of the Witwatersrand Johannesburg South Africa

## Abstract

Interaction with a lay referral system (informal networks that provide health advice during illness) influences the treatment‐seeking decisions of individuals with perceived healthcare needs. We examined how this occurred in urban slums in Nigeria with scarce formal healthcare and unstable social networks. Using existing theories of social networks and lay referral, we examined the use of these systems for treatment‐seeking decisions in slums, including the use of digital technologies. We interviewed 30 adults (aged 18–64) of diverse age, gender, network size and use of digital technologies for health advice in two Nigerian slums. We analysed the data using a thematic inductive–deductive approach. Lay referral was multidimensional: discussion of illness during daily bonding, social demonstration of self and purposeful exchange of support. People limited lay referrals to a few family members and friends, avoiding wider interactions due to mistrust. Use of online sources was scarce due to limited access to smart devices and low digital health literacy. Lay referral motivated timely care seeking but also facilitated unhelpful advice. Slum residents were agentic in their use of their lay referral system. The effectiveness of their agency may be improved if trusted and reliable health advice sources are available in addition to their social network.

## 1 Introduction

### 1.1 Navigating the Lay Referral System: Traditional and Digital Pathways

Interaction with lay referral systems is a common mechanism affecting health‐related decisions. The lay referral system consists of social connections (e.g., family members) and other sources of communication and interaction (e.g., mothers forum) whom individuals turn to in their daily lives to address their health issues (Freidson 1988). The scope of the discussions can cover a wide range of topics including prevention and rehabilitation. However, in the context of this study, we refer to interactions with lay referral networks when individuals encounter symptoms of illness or health concerns for which they need care or advice. This is also known as lay consultation. When people encounter illnesses or health concerns that are new, unusual, disrupting their daily lives, urgent or severe they tend to turn to their lay referral system for discussion and the interaction can influence treatment decisions (Queenan et al. 2018).

Traditionally, the lay referral system consists of proximate social network members. People begin interactions with the contacts within their immediate environment and then extend to farther‐located networks (Freidson 1988). Family members are the most consulted across different social contexts (Mwaka et al. 2021; Reeder et al. 2017; Queenan et al. 2018; D. Smith et al. 2020). This is unsurprising given the physical proximity, emotional connections, and family obligations to care for each other (Defossez 2020). Other networks include friends, neighbours, colleagues and community members (D. Smith et al. 2020; Amoah, Edusei, and Amuzu 2018). People who have had similar illness experiences are consulted for guidance, and their experiences are used to compare and assess a condition (Cooper and Gilbert 2017; Defossez 2020).

Digital communication technologies enable consultations with the lay referral system to take place remotely and provide the opportunity to expand personal networks (Loignon et al. 2022). Internet sources such as websites, online discussion forums and social networking sites have become components of people's lay referral system (Defossez 2020; Loignon et al. 2022). Some websites and apps have AI‐driven interactive elements (Chatbots) that give a sense of consulting with a person (Javaid, Haleem, and Singh 2023). These remote sources offer opportunities for people to look up information/advice, engage in discussions, and read other people's exchanges. Use of digital sources may happen concurrently, before or after consultation with personal network members (Borlescu 2016). A recent study found that parents sought health advice regarding infant care from an informal online discussion forum before consulting friends and family members (Loignon et al. 2022). Digital sources can provide people with access to a wider range of advice, and support individuals with poor access to physical networks or with illnesses they do not wish to share with their physical networks (Bohn et al. 2014; Defossez 2020).

However, the ability to access and use digital technology for health varies, influenced by level of education and contextual factors such as socioeconomic status, availability of social support for its use and digital infrastructure (Lythreatis, Singh, and El‐Kassar 2022). Individuals lacking digital and literacy skills to navigate the internet for essential health advice face exclusion (Defossez 2020). People using online discussion forums can encounter contradictory advice causing confusion (Defossez 2020; Loignon et al. 2022). Using online sources can have consequences on health behaviours including increased or decreased help‐seeking (Mueller et al. 2017).

A key concern remains how lay consultation, whether traditional, online or both impacts individuals' treatment‐seeking decisions. Informal conversations with social networks about health are part of the everyday taken‐for‐granted activities and can be difficult to report (Garfinkel 1964). Yet these interactions can influence how and whether an individual seeks healthcare. Network members assess symptoms, influencing how the symptom bearers perceive their urgency and severity (Queenan et al. 2018). They can share advice about medications and home treatments and encourage people experiencing illnesses to seek formal or informal care (D. Smith et al. 2020; Reeder et al. 2017). Network members can offer emotional support (e.g., listening, caring and sympathising) for coping with illnesses, and practical support (e.g., financial support) to facilitate use of health services (Mwaka et al. 2021).

However, there are negative consequences of lay consultation. Network members may transmit advice, norms, and values that may discourage formal healthcare‐seeking (D. Smith et al. 2020). Network members sharing prescription advice from health providers with others contribute to self‐medication (Rodrigues 2020). Informal health interactions may be intrusive and stigmatising, creating negative feelings among symptom bearers (Mwaka et al. 2021).

The social support gained from lay consultants may not be equally distributed to individuals within networks (Lubbers, Small, and García 2020). This can cause negative emotions and affect the coping capacities of those sidelined (Cooper and Gilbert 2017). Overreliance on networks can make those needing support feel inadequate contributing to relationship dissatisfaction (Defossez 2020). Advice and assessments from network members may be ineffective in helping people understand their symptoms or decide on treatment actions (Price et al. 2021).

### 1.2 Slum Living and Health Challenges

This study explores lay consultation within slum contexts. The term ‘slum’ is considered pejorative by some, with ‘informal settlement’ proposed as a less negative alternative (Zerbo, Delgado, and González 2020). However, there are key differences between the two terms (Ezeh et al. 2017). According to the United Nations Human Settlement Programme (UN‐Habitat), slums are units lacking access to improved water, sanitation, sufficient living space, durability of housing, and secure tenure (UN‐Habitat 2020). Informal settlement on the other hand connotes illegal occupancy of land (Zerbo, Delgado, and González 2020). In Nigeria, not all slums are informal settlements. Some slums are legally occupied structures that have deteriorated due to a lack of proper maintenance (Fourchard 2003). This study focuses on communities categorised as slums based on the indicators provided by the UN‐Habitat. Our usage of the term is also in line with the broader project within which this current project was based (Improving Health in Slums Collaborative 2019).

Slums are ‘one of the most enduring faces of poverty, inequality, exclusion and deprivation’ (UN‐Habitat 2020, 25). Globally, one in eight persons resides in slums, and 60% of the urban population in African countries including Nigeria live in slums (UN‐Habitat 2020). Slum dwellers struggle with inadequate access to basic social infrastructures and amenities, and are highly vulnerable to diseases (Ezeh et al. 2017). Understanding slum health contributes to achieving the United Nations Sustainable Development Goal to ‘make cities and human settlements inclusive, safe, resilient and sustainable’ (UN‐Habitat 2020).

A major challenge faced by slum dwellers in low and middle‐income countries is poor access to comprehensive formal healthcare (Ezeh et al. 2017). In slums within Nigeria, there is a scarcity of formal healthcare services, a scarcity of healthcare workers, and difficulties in affording available private healthcare (Onwujekwe et al. 2022). There is heavy reliance on informal healthcare providers without professional registration particularly patent medicine vendors (PMVs) and community health workers (CHWs) (Onwujekwe et al. 2022). A previous systematic review and an empirical survey by the authors found that lay referral networks are significantly involved in treatment decisions in slums (Onuegbu et al. 2021; Onuegbu, Harlock, and Griffiths 2023). Most people spoke to at least one lay consultant in a recent illness or health concern requiring care or advice, although most reported that the conversations were casual. The lay referral systems consisted close of family members, friends and neighbours who were contacted via face‐to‐face interaction (Onuegbu, Harlock, and Griffiths 2023).

However, these studies are limited in that they do not explore the process of interaction with the lay referral system. Social relationships and interactions can be complex due to contextual factors in the slums. Social relationships and interactions in slums are shaped by co‐existence in intimately shared physical environments, heterogeneity, frequent migration of people and possible eviction by city authorities (van de Vijver et al. 2015; Sarkar 2020). Social exchange and reciprocity are difficult to maintain as people with some resources risk being drained by helping those with fewer resources (Greif and Dodoo 2015). These factors contribute to relationships being transient and unstable. There is a poor understanding of the process of interacting with the lay referral systems within the complexities of slum life, the content of the interaction and how this is used to inform personal treatment decisions—gaps that this study aims to fill.

Digital technologies have become an important component of people's lay referral system, and their access has become widespread in Africa; however, they are still limited in low‐socioeconomic groups (Silver and Johnson 2018). These groups are less likely to have the economic means to own smart devices or the literacy to access health advice from the internet (Garner, Sudia, and Rachaprolu 2018). Corroborating this, the previous survey in the slums undertaken by the authors found that 95% of the respondents reported that they had never sought health advice from the internet (Onuegbu, Harlock, and Griffiths 2023). Despite this, it is important to understand how digital technologies are used by the few who adopted them as part of their wider information gathering for treatment decisions. This qualitative study will explore in more depth the reasons why there is limited use of digital technologies in lay referrals in the slums, to add to existing knowledge from our survey and previous works.

### 1.3 Theoretical Approach: Social Network Theory and Lay Referral Theory

Social network theory places its central focus on the interactions, ties, and relationships of social actors within a network (Borgatti and Ofem 2010). This is in contrast with individualism which focuses on micro‐level individual actions and structuralism (e.g., functionalism and Marxism) focusing on large social entities (Crossley 2015). Networks are at the meso level, mediating between both individual actions and social structures (Gamper 2022). Individuals are shaped by the characteristics, interactions and culture within their networks (Crossley 2015). Networks and their pattern of interactions shape the overall social structures (Gamper 2022).

In the health domain, social network theory provides a framework for understanding the link between social networks and personal health outcomes (K. P. Smith and Christakis 2008). As explained by Freidson:

The organization of people into families and other kin groups, neighbourhoods, work groups, cliques and the likes…operates to enforce particular views of illness and its treatment irrespective of the views of the isolated individuals within it.

(Freidson 1988, 289)

There are three broad applications of the social network theory (Emirbayer and Goodwin 1994). The first is structural determinism which interprets human actions as a result of network attributes. This has been criticised for paying insufficient attention to individual agency in establishing, maintaining and engaging social ties (Burt, Kilduff, and Tasselli 2013) and for ignoring the role of network culture (Gamper 2022). The second, structural instrumentalism, emphasises an individual’s actions and agency within the network, with a strong focus on social actors, but is criticised for not sufficiently recognising that networks create opportunities or constraints for individual action (Crossley 2015). Third is structural constructionism which explains human action as a product of agency, culture and social structure, highlighting their interconnectedness. Culture is created and sustained through social interaction within networks, and in turn, shapes individual actions (Crossley 2015).

An example of structural constructionism is Friedson's lay referral theory. Freidson argues that the decision to seek care is organised within consultation with a lay referral system (Freidson 1988). Individuals experiencing illness would first consult lay consultants (individuals within their networks) within their families and progressively engage with more distant others until a health professional is reached. The lay referral system influences treatment‐seeking decisions through two mechanisms: (1) the nature of relationships between individuals and lay consultants and (2) the culture or knowledge in the networks (Freidson 1988). Individuals embedded in close relations with people who uphold folk medicine and practices different from formal medical practice are likely to defer formal treatment. In contrast, those in loose relationships with lay consultants who share and promote the use of formal healthcare tend to seek formal healthcare (Freidson 1988).

Freidson's theory is criticised for projecting a functionalist view of the lay referral system as performing functions for the overall management of health, with insufficient emphasis on the personal agency of symptom bearers and on how lay consultation can be used to control or discriminate against others (Hiscock 2013). Furthermore, Friedson's theory was introduced in the 1970s when there was limited digital communication. As discussed in the introduction, digital communication facilitates interaction with a range of new types of digital agents, and it would be important to explore how this applies to lay consultation.

This paper examines the experiences of the lay referral system in the slums of Nigeria through the lens of structural constructionism. The aim is to identify how personal agency, environmental factors and network culture intersect in the use of lay referral system.

### 1.4 Research Aim

To understand how and why people living in urban slums of Nigeria use lay referral systems for treatment‐seeking decisions amongst people, including the use of digital communication/online sources within these networks.

## 2 Methods

### 2.1 Study Design

This qualitative study explores the experiences of using lay referral system in slums of Nigeria. Building upon our previous survey which mapped and described the structural dimensions of the lay referral system (Onuegbu, Harlock, and Griffiths 2023), this study explores experiences of use of the networks in more depth, using in‐depth interviews.

The previous survey was conducted amongst community members aged 18–64 (working population) residing in two slum communities in Oyo state, Nigeria (Onuegbu, Harlock, and Griffiths 2023). We refer to the communities as Community A and Community B to protect their identities. During the survey, participants were asked if they were happy to be invited for further in‐depth interviews. Those who responded positively were asked to provide their phone numbers for contact purposes.

The study was planned before the COVID‐19 pandemic when it was expected we would conduct face‐to‐face interviews. With COVID‐19, we re‐planned the study so we could undertake interviews by phone. Interviews were conducted from January 2021 to August 2021.

### 2.2 Ethics

Participants provided oral informed consent at the start of the interview, which was not audio‐recorded. They consented to understand the study details, the voluntariness of their participation and their right to withdraw from the research at any time. They also provided consent for the audio recording of the interviews, the use of their data for future research and their participation in the study.

### 2.3 Sampling and Recruitment

From our survey results we knew our participants age, gender, lay referral network size, use of online sources of health advice and reasons for seeing health advice through their lay referral network.

We chose to stratify our sampling for this interview study by use of online sources of health advice and size of networks to ensure diversity of participants on these dimensions. We aimed for diversity of age/gender across the whole sample. From our survey results, we know that 40% of survey respondents engaged with their lay referral network about infection, mostly malaria and 60% had other concerns including headaches, general pain and weakness, specific musculoskeletal pain and gastrointestinal and abdominal issues (Onuegbu, Harlock, and Griffiths 2023). Due to the relatively small sample size of our interview study, we did not stratify participants based on the type of illness.

We grouped participants into five categories as shown in Table 1.

**TABLE 1:** Participant categories for the interviews.

Categories	Description
One	Respondents who used online sources of health advice and interacted with one or two lay consultants
Two to four	Respondents who did not use online sources of health advice and had no interaction with a lay consultant (three), interacted with one or two lay consultants (four) or spoke to three or more lay consultants (five)
Five	Respondents selected for interviews to explore emerging issues from interviews with those in categories one through four

We randomly selected participants from the five categories listed in Table 1. Subsequently, we contacted 2–3 potential participants at a time within each category and monitored responses. As we gained participants, we oversampled to ensure each category included both male and female participants from diverse age groups (18–29, 30–49 and 50–64), recognising that they may have different experiences of using lay consultants (Das et al. 2018).

We phoned potential participants to recruit and schedule them for an interview. Up to three attempts were made to contact each participant before they were considered non‐responders.

### 2.4 Interview Process

We used a semi‐structured interview guide, informed by theory, existing literature (Hiscock 2013) and our survey results (Onuegbu, Harlock, and Griffiths 2023). The guide used a reverse funnel approach to ground the discussion in reality (Morgan 2012). We initially asked about experiences of interacting with lay social network members and using online lay advice sources about a recent illness/health concern before asking about more general experiences. We explored how people chose lay referral system, why they used them, how people made decisions using lay referrals and how online sources were incorporated into the process. The interview guide was translated to local languages, Yoruba and Hausa, by a local translator and checked by another language expert for accuracy. The interviews were conducted by a local research assistant (RA) who was proficient in both languages. The RA was not familiar with doing phone interviews, so J.H. and F.G. guided C.O. to train the RA. C.O. held repeated mock interviews with the RA in English to ensure that the guide was consistently administered. The RA kept field notes about recruitment and interview processes. C.O. debriefed the RA after each interview and reviewed the field notes. The debrief was useful for monitoring and reviewing the interviewing techniques, rephrasing questions that were not clear after initial interviews and checking understanding of interview data.

### 2.5 Data Analysis

All interviews were audio‐recorded, transcribed verbatim and translated into English by the RA. The transcripts were transferred into NVivo software for analysis. The analyses followed an inductive–deductive approach adapted from Thomas and Harden (2008). This involved using the research questions to guide the coding and analysis, but allowing codes and themes to emerge inductively from the data, following three broad steps. First, C.O. read the first 10 transcripts, F.G. and J.H. each read five of the transcripts. We met in pairs (C.O. and F.G. and C.O. and J.H.) and subsequently together to discuss our initial analysis ideas and review the guide and technique for later interviews. C.O. read the rest of the transcripts. Second, C.O. coded the interviews line‐by‐line or collections of sentences to develop a list of codes, examined the codes for similarities and differences, grouped them into descriptive themes and then reviewed the descriptive themes to identify analytical themes. F.G. and J.H. reviewed each of the stages and provided their feedback, and we met as a team at each stage to review and agree on the outputs. Finally, C.O. reviewed each theme to check for variations in participants' responses.

## 3 Results

Thirty participants were interviewed. We contacted a total of 93 persons: 56 could not be reached via the phone numbers they provided during the survey, five declined participation without giving reasons and two withdrew after starting the interview. Most of the final participants (24 out of the 30) were recruited from Community B. There was community unrest in Community A between the survey and the interviews, resulting in several individuals leaving the community. Consequently, contacting participants for the interviews became challenging as many were unavailable or unable to participate.

Our final sample had equal numbers of participants (6) representing each of the five categories (see Table 1). Participants within each category were distributed across age groups and genders. The overall sample consisted of slightly more men (16/30) than women (14/30). Additionally, there were slightly more participants in the 18–29 (11/30) age category compared to those in the 30–49 (10/30) and 50–64 (9/30) categories.

### 3.1 Main Themes

We identified the following six main themes from our analysis of experiences of use of lay referral system: (1) intentions, motivations and outcomes of engaging with a lay referral system, (2) use of closely‐knit and immediate network members, (3) limited use of digital technologies for lay referral, (4) deciding on how to act on advice from others, (5) choosing not to disclose illness and health concerns or see advice from lay referral system and (6) giving lay advice and support to others.

### 3.2 Intentions, Motivations and Perceived Outcomes of Engaging With a Lay Referral System

We identified three primary intentions that characterised why people discussed a health concern or an illness with their lay referral system: unintentional reporting about personal health during day‐to‐day interactions, direct solicitation for support, and indirect seeking of support. Most participants described their conversations as casual discussions in which they mentioned an illness/health concern to someone in their network without any specific intent to seek support. In such cases, participants mentioned speaking to someone who was accessible or nearby at the time or because it was considered a normal practice as explained by a participant:

I just told mummy landlord (*referring to landlady*) because we both sleep and wake up in the same house. For my family, I had to tell them. Not that the issue was something I could not handle myself.

(Category 5/F/18–29)

Some of the conversations happened between neighbours who noticed a change in their neighbour's appearance or behaviour.

My neighbours checked on me when they noticed that I was at home – because it was unusual to see me at home during the day. They came and knocked on the door to ask ‘Hope all is well, we see you at home?’. I told them that I was a little ill, and they asked me if I had taken drugs or injections.

(Category 5/M/30–49)

About half of the participants either consulted their lay referral system or expressed an intention to do so, seeking various forms of support. Advice was sought about causes of symptoms/illness, advice on medicines, alternative medications or home remedies based on the network members past experiences with similar symptoms. Some people sought financial support to care for themselves during an illness. A few participants wanted their network members to empathise with them and provide hands‐on assistance:

Ah, I told her so I can be assisted with what I cannot do, maybe I want to sweep a place, and I cannot sweep it, I will say *oya* sweep here for me, I have backache, I cannot sweep it.

(Category 4/F/50–64)

One‐fifth of the participants reported engaging in informal health conversations wherein they expressed health concerns to a network member without directly seeking support, yet still expected to receive some form of assistance or support. Both men and women reported this behaviour, but there were slightly more men than women. For instance, one man giving his reasons for discussing a health concern with his wife said:

I just wanted her to know, because once she does, she would definitely have something to say.

(Category 4/M/30–49)

Participants consistently reported that when they spoke to their network members, irrespective of their initial intention, the network members offered various forms of advice or suggestions. The advice encompassed labelling the symptoms, suggesting potential causes and treatment recommendations including home remedies and formal and informal healthcare. Sometimes network members volunteered health advice without being asked. For instance, a daughter noted the remedies received from her parents:

When I discovered a twitch in my feet…and I was staggering while walking, my parents noticed and said it was due shortage of blood. They said I should buy Ugwu (*spinach*), malt and milk. That I should mix it and drink it.

(Category 4/F/30–49)

Participants had mixed reviews about the advice received from their lay consultants. About one‐third of the participants thought they were useful as they felt better after following the advice. One participant said they received useful and non‐useful advice from the same network members. A few participants reported negative experiences following lay advice about medicines from lay consultants. For instance, a participant shared an incident in which a neighbour recommended taking a 60 mg dose of tramadol when she complained of feeling weak. Following the usage, the participant felt ‘dizzy, fainted…and landed in the hospital’, where she was informed by healthcare professionals that the use of tramadol was prohibited in Nigeria (Category 4/F/30–49).

Aside from advice, other forms of support reported included the provision of medicines or herbal remedies, being accompanied to the health provider, assistance in contacting health workers and the offering of prayers and reassurance.

### 3.3 Use of Closely‐Knit and Immediate Network Members

We found that participants lay referral networks comprised connections characterised by emotional connection, proximity, trust and shared expectation of care. These were family, friends and some neighbours. Family members were usually the first or only persons consulted, and the specific family members used varied based on the circumstances of the participants. For instance, both married men and women tended to speak to their spouses, younger participants spoke to their parents, especially their mothers, and older participants spoke to their adult children. Family members noticed or detected changes in each other's health and provided needed support:

I told my wife because, you know, she is my defence minister…If something happens to me, she would be the first to know, she knows what usually happens to me and what she uses for me that makes my symptoms resolve. She knows who to call, and the kind of medicine that would make my body okay.

(Category /5/M/30–49)

A small number of participants discussed a health concern/illness with selected friends and neighbours—both men and women. These were usually people in their immediate environment, however proximity alone did not drive the consultation. Important factors considered were perceived knowledge of the network member, mutual respect, experience with a similar health concern, intimate connection, reciprocity and trust that the person would not divulge private information they share with them.

I have friends, and I also have neighbourhood sisters. I receive advice from them very well, and they do not look at me as a small girl to them, they also seek advice from me. So, we help each other.

(Category 5/F/18–29)

### 3.4 Limited Use of Digital Technologies for Lay Referral

Participants mainly relied on face‐to‐face interactions with proximate network members, while only six out of 30 engaged in phone conversations or chats with distant network members or consulted online sources of health advice. These participants sometimes reported a health concern during casual catch‐ups. However, there were a few instances where participants called a network member to inform them about an emerging health concern, as explained by a woman in the example below:

I couldn’t taste anything…and I couldn’t smell anything…meanwhile, I used to smell very well. My husband was on night duty…I called him on the phone to tell him about my experience. He now said I should allow him to return (*home*). So I explained to him in details when he got back (*home*)…He said I should go to the hospital, the one that is close to us so as to make complaints so that they can give me medications to use.

(Category 3/F/30–49)

Only three participants actively used the internet to inform their decisions about a health concern. A participant used Google to look up and label his symptoms and inform a change in his treatment choice:

I just typed my symptoms…What I saw was that they (*the internet*) said I perhaps had typhoid. I checked (*the internet*)…after I had gone to buy medication at the chemist. So, it was after checking that I realized that it was beyond going to the chemist and was a matter of the hospital.

(Category 4/M/18–29)

Most accounts on the use of the internet were about finding or sharing general health information about health and not seeking advice about treatment‐seeking decisions. Network members belonging to online groups such as Facebook or WhatsApp groups where health advice was shared, passed it on to others in their networks who might find it beneficial.

Since my big phone (*referring to smartphone*) has been missing, I do not really check (for health advice). Anyone that our daddy *(referring to her husband)* has on the phone‐ the one he sees on the internet because he cannot do without checking it, he usually tells me. I will then sit down and think about it…I make use of the ones I deem fit.

(Category 3/F/30–49)

There were barriers to using online sources of advice. A common one was the lack of smart devices. Several participants mentioned being unable to afford the devices, or having other immediate needs. One man explained that paying for his children's education was more of a priority ‘than the touch screen phones’ (Category 4/M/30–49).

One‐third of the participants cited poor digital literacy or poor knowledge of searching for health advice online as reasons for not accessing it. Older participants in particular, were more inclined to express this concern, perceiving the internet as primarily used by younger individuals.

I have never checked (*the internet for health advice to manage a symptom*) before, it’s my children who use Google. Sometimes she has an assignment from school and she wants to check it, she is the one who uses it.

(Category 4/F/30–49)

A few participants knew how to operate smart devices and used the internet for other purposes, but were not aware they could obtain targeted health advice online:

If not for the way you mentioned it now, I did not know they check for health issues online. I use it for checking stories, joining groups, and commenting. So, I have not even come across health groups not to talk of joining.

(Category 5/F/18–29).

One barrier to online health information seeking was the medical jargon used in delivering medical information. One woman explained that online health advice on websites were often written in technical terms: ‘It is not clear, only medical students can understand it’ (Cat 1/F/18–29).

Few participants noted that they were not accustomed to using online health advice sources in assessing emerging symptoms. For instance, a man expressed that checking online for health concerns that require immediate intervention was impractical:

If I am not feeling fine now, you cannot expect me to carry my phone and search for what is wrong with me. Rather, when I am sound and healthy, I can check for what is wrong.

(Category 1/M/30–49)

### 3.5 Deciding on How to Act on Advice From the Lay Referral System

Participants actively chose how to engage with advice and suggestions offered by others. They decided on whether the opinions of others were useful or not. For instance, a man classified advice from others as ‘good, bad or non‐substantial’ (Cat 3/M/50–64). Participants explained that they had personal approaches and strategies to address their health concerns and illnesses, which they used to filter advice and suggestions from others.

In any advice they give me, my opinion matters a lot. When somebody gives me advice, I feel that is their own opinion. If I feel that the advice is okay, then I may follow up, but if the advice is not okay by me, then I will not do it, and that is it.

(Category 1/M/30–49)

Some participants were critical of health advice or information from network members without medical expertise. They recognised that the opinions are likely to be subjective and questioned the source and credibility of such health advice:

If someone who is not a doctor or nurse says something about health, I may not do it because I will be wondering how they got the information. Like ‘How do you know what you are saying?’

(Category 4/F/18–29)

Some participants consulted multiple persons or perceived experts to determine the credibility of an advice they had received from a network member. Getting similar advice from multiple lay network members gave people some confidence. Some confirmed with patent medicine vendors (unregistered medicine sellers) particularly regarding advice about medicines. Only a few participants confirmed with formal medical care providers such as medical doctors as this required some form of payment for consultation. Some seeking care from the health practitioners’ verified advice received from network members.

There were two instances where participants said they had no choice but to utilise advice from network members. In one instance, a middle‐aged woman said she was too ill so her husband sought advice and made the decisions on her behalf. In another instance, a young woman said she followed her husband's advice to seek care from a particular provider because he had ‘authority’ in their household as he paid for healthcare.

### 3.6 Choosing Not to Disclose Illness and Health Concerns or Seek Advice From a Lay Referral System

People exercised agency by deciding not to disclose their illness/health concerns or seek advice from others. The decision was linked to how people perceived their health needs or relationships with others. Several participants reported experiencing familiar symptoms, which they often managed independently using herbal remedies or obtaining medicines from PMVs, thereby reducing the need for advice from the lay referral system.

Few participants did not seek advice from lay others because they preferred and had access to formal health care providers. For instance, one man who accessed formal health care insurance through his employer explained his preference for formal health advice:

I cannot have a health issue now and then consult a farmer to explain to him… I cannot have a headache and meet with a market woman… The Hospital is the best place to get advice concerning that. You may now have to run tests to know what exactly happened.

(Category 1/M/30–49)

A few participants did not discuss a health concern they were experiencing with others because they did not want to bother them or create a sense that they required attention. In one case, a respondent said they did not appreciate being called and checked upon frequently by network members because they were unwell and were more interested in getting solutions to the problem as soon as possible.

However, about half of the participants highlighted mistrust towards some neighbours as a reason for non‐disclosure. A common notion amongst the participants was that neighbours would gossip, mock or deliberately offer them negative advice:

I do not seek advice because I realised that once I discuss things like that with neighbours, it is like I am doing more harm to myself. Because it is not every frog that lies on their stomach that has a stomach ache (*meaning not everyone has good intentions for you*)…I might ask a question about stomach ache, and in their minds, they would say, ‘Oh he is just having stomach ache, I hope he will soon have a headache’.

(Category 2/M/18–29)

People who distrusted their neighbours avoided speaking to them about their experience, minimised their condition when discussing it with them or anonymised themselves when asking neighbours for advice about their experience.

### 3.7 Giving Lay Advice and Support to Others

About a third of participants talked about giving advice and assistance to other network members. Some said that network members approached them for health‐related advice because they possessed physical health attributes and social qualities that others admired. For instance, one man said network members admired and approached him for advice because they were rarely sick:

They usually wonder why, despite how much I labour (because I do not like being idle) I do not usually fall sick. They ask me, ‘What is the secret to your well‐being?’ So I tell them: ‘You, go do this, go do this’.

(Category 5/M/50–64)

Some thought others approached them because they had knowledge or experience of health issues, empathy and compassion. Participants expressed that they were willing to support others in their networks with fewer resources:

If I have someone whose situation is not as good as mine, I do help in my way.

(Category 5/F/50–64)

However, it was important to participants that their advice or support was appreciated, as this would determine whether they provided continued support:

If I advise someone today and the person does not heed, if the person comes back at another time, I will not respond because they didn't turn up on the previous advice I gave… For instance, you have stomach pain and I ask you to use Buscopan (*a medication*), but you used paracetamol; please can the pain resolve?

(Category /5/F/18–29)

Giving support to others was a strategic way of investing in networks with the hope of receiving their support if needed in the future. For example, one woman said they were open to helping despite not having enough resources because:

If I help someone today, I do not know who will help me tomorrow nor who will help my children.

(Category /5/F/18–29)

However, a few participants did not advise others in the networks because their busy schedules did not allow them to socialise much, limiting opportunities for such interactions, or because they thought people would not trust their advice. Those who cited the latter reason were referring to neighbours with whom they had a complex relationship. They avoided telling these neighbours about lay therapies or referring them to particular health providers because the neighbours might suspect they were providing misleading advice.

## 4 Discussion

This study aimed to explore the experiences of using the lay referral system in the slums of Nigeria. The utilisation of the lay referral system is influenced by the interplay of socio‐environmental factors, complex social relationships and interactions and personal agency within the slum context. Participants engaged with a small and closely knit group, and this was linked to mistrust for extensive networks. There was low usage of internet sources of advice due to poor digital access, low digital literacy and a culture of talking to immediate networks. Advice and support shared by network members were sometimes useful and sometimes not. People expressed a strong sense of agency in speaking about their health to others, choosing when to initiate such conversations, whom to talk to or avoid, and how to use advice from others.

The study findings highlight the role of cultural norms on lay referral behaviours. Gendered patterns were observed in the use of lay referral systems, with mothers being more frequently consulted for health advice compared to fathers and husbands. This aligns with the common expectation that mothers take an active role in gathering and providing health information as part of their roles in being the managers and experts of health of the family (Das et al. 2018). However, the use of few immediate network members mainly family members in the study may have hampered observing this difference.

The study underscores the importance of relational dynamics and socio‐cultural contexts within lay referral networks. Established norms of reciprocity and interaction determine whom individuals approach for advice, support and solidarity as well as whom to avoid or distance themselves from. Trust was also key in the use of lay referral networks. Proximity facilitates access to network members, however trust in their capacity to offer valid information and care and discreetly handle people's health‐related information partly drives lay consultation (Perry and Pescosolido 2015). This is illustrated in the use or avoidance of neighbours depending on presence of trust and norms of reciprocity.

In slum settings, where residents live in close proximity and share common challenges, there is an expectation of community interdependence, and our study show some aspects of this. However, this expectation may not always be fulfilled due to factors such as lack of a cohesive community identity, poverty and insecure tenure (Das et al. 2018; Sunikka‐Blank, Bardhan, and Haque 2019). For example, a study conducted in Indian slums revealed that residents refrained from seeking or providing advice to neighbours to avoid burdening others or becoming burdened themselves (Das et al. 2018).

Participants in our study reported poor digital access and low digital literacy preventing them from accessing websites and online groups. This implies that people could mainly access from their immediate networks of people facing similar realities, reinforcing inequalities in these groups (Pew Research Center 2016). Similar challenges have been noted by other studies in slums in Africa and Asia (Harris et al. 2021; Nsor‐Anabiah, Udunwa, and Malathi 2019).

Our study highlights the importance of human agents in social networks and supports the critiques of network studies for paying insufficient attention to human agency (Burt, Kilduff, and Tasselli 2013). Indeed, people belong to networks that might provide or limit opportunities, but they choose and determine how to act on the opportunities (Burt, Kilduff, and Tasselli 2013). Our study demonstrated that people chose whom to consult for health advice and how to use the advice.

Our study resonates with other research undertaken in slums that demonstrates that people living in the slums are agentic, even though the agency might be small‐scaled and less visible given the structural constraints they live in (Willan et al. 2020; Gillespie et al. 2021). Slum dwellers are faced with extreme resource constraints contributing to reliance on lay social networks (Mpanje, Gibbons, and Mcdermott 2018; van der Heijden et al. 2019). However, our study demonstrated that despite these constraints, people still had opportunities to exercise agency in how and when they chose to elicit and act advice from lay referral networks.

A major strength of this study is that we were able to interview adult slum dwellers aged 18–64, which is the working‐age population in Nigeria. Slum dwellers are generally hard to reach, and adult dwellers are even less likely to be accessible due to busy work schedules (van de Vijver et al. 2015). This study makes a major contribution by eliciting information on how adults manage their treatment decisions through lay consultation. Our sample was also diverse, encompassing different age groups and genders; however, one of the two slums had more representation.

As the interviews were conducted during the COVID‐19 pandemic, we provided clear information and reassurance regarding the focus of our study and to distinguish it from COVID‐19 surveillance or providing health education (Khalil et al. 2021). Admitting to having the symptoms could have meant being isolated in a government facility, as per the Nigerian public health directive at the time (Nigeria Centre for Disease Control and Prevention 2020). For instance, a few participants in our study needed clarification that the interviews were not aimed at identifying persons with recent COVID‐19 symptom experiences.

Furthermore, it is important to note how the use of phone interviews affected data collection. Use of mobile phones for qualitative interviews, while common in high‐income countries and settings, is relatively new in LMICs (Reñosa et al. 2021). Remote data collection limited our sample to people with access to mobile phones and who had not changed their phone numbers since the survey. This is likely to have excluded underserved groups (Reñosa et al. 2021). Our research assistant and participants were not used to this form of data collection, which may have resulted in insufficient rapport for rich discussions (Horsfall et al. 2021). To mitigate this, we used role play in training the research assistant and reviewed the interview technique throughout data collection to optimise data quality.

## 5 Conclusions

The study demonstrates an interplay of environmental factors, personal agency and network culture in the use of lay referral system. Despite living in the extreme resource constraints that drive reliance on lay referral networks for resources to cope with illnesses, people in slums of Nigeria exercised a strong sense of agency in how they engaged with the system and applied their advice. Small networks of lay consultants were involved in how people perceived, evaluated and responded to illnesses. Conversations with lay referral systems are largely taken for granted, but they facilitated the transfer of various forms of social support, some useful and some not.

We suggest that if formal trusted sources of advice were available, slum dwellers are likely to deploy this same strong agency to seek and use this advice. This could be in the form of a health advice website, run by a trusted organisation/government body that would, amongst other things, guide people to self‐manage symptoms that do not require medical consultation and signpost people to the formal medical consultation when needed. Digital devices could be made available in the community, for example at health centres, for use by people without access to smart devices. Technical helpers at the health centres (Ahmed et al. 2015) can guide people who lack digital literacy on operating the devices without providing them with health advice.

## Author Contributions

**Chinwe Onuegbu:** conceptualization (lead), data curation (lead), investigation (lead), methodology (lead), project administration (lead), visualization (lead), writing—original draft (lead), writing—review and editing (lead). **Jenny Harlock:** conceptualization (supporting), investigation (supporting), methodology (supporting), writing—review and editing (equal). **Frances Griffiths:** conceptualization (supporting), investigation (supporting), methodology (supporting), writing—review and editing (equal).

## Ethics Statement

Ethical approval was obtained from the Biomedical and Scientific Research Ethics Sub‐Committee, University of Warwick, UK (BSREC 17/19–20 AM01) and the Research Ethics Committee of the Oyo State Ministry of Health (AD13/479/2035).

## Consent

All participants provided informed consent to participate before taking part in the study.

## Conflicts of Interest

The authors declare no conflicts of interest.

## Data Availability

The datasets generated and analysed during the study are not publicly available but may be available from the corresponding author on reasonable request.

## References

[shil13863-bib-0001] Ahmed, A. , A. Rebeiro‐hargrave , Y. Nohara , et al. 2015. “Portable Health Clinic: A Telehealthcare System for Unreached Communities.” In Smart Sensors and Systems, edited by Y. L. Lin , C. M. Kyung , H. Yasuura , and Y. Liu . Cham: Springer. 10.1007/978-3-319-14711-6_18.

[shil13863-bib-0002] Amoah, P. A. , J. Edusei , and D. Amuzu . 2018. “Social Networks and Health: Understanding the Nuances of Healthcare Access Between Urban and Rural Populations.” International Journal of Environmental Research and Public Health 15, no. 5: 973. 10.3390/ijerph15050973. 29757256 PMC5982012

[shil13863-bib-0003] Bohn, A. , C. Buchta , K. Hornik , and P. Mair . 2014. “Making Friends and Communicating on Facebook: Implications for the Access to Social Capital.” Social Networks 37: 29–41. 10.1016/j.socnet.2013.11.003.

[shil13863-bib-0004] Borgatti, S. P. , and B. Ofem . 2010. “Social Network Theory and Analysis.” In The Ties of Change: Social Network Theory and Application in Education, edited by A. J. Daly , 17–29. Cambridge: Harvard Press.

[shil13863-bib-0005] Borlescu, A. M. 2016. “Representations of Self‐Care Treatment Practices in Patients’ and Doctors’ Discourses: Between Non‐Compliance and Agency.” Transylvanian Review 25: 45–60.

[shil13863-bib-0006] Burt, R. S. , M. Kilduff , and S. Tasselli . 2013. “Social Network Analysis: Foundations and Frontiers on Advantage.” Annual Review of Psychology 64, no. 1: 527–547. 10.1146/annurev-psych-113011-143828. 23282056

[shil13863-bib-0007] Cooper, S. , and L. Gilbert . 2017. “The Role of ‘Social Support’ in the Experience of Fibromyalgia–Narratives From South Africa.” Health and Social Care in the Community 25, no. 3: 1021–1030. 10.1111/hsc.12403. 27782344

[shil13863-bib-0008] Crossley, N. 2015. “Relational Sociology and Culture: A Preliminary Framework.” International Review of Sociology 25, no. 1: 65–85. 10.1080/03906701.2014.997965.

[shil13863-bib-0009] Das, M. , F. Angeli , A. J. Krumeich , and O. C. Van Schayck . 2018. “Patterns of Illness Disclosure Among Indian Slum Dwellers: A Qualitative Study.” BMC International Health and Human Rights 18, no. 1: 3. 10.1186/s12914-018-0142-x. 29338708 PMC5771001

[shil13863-bib-0010] Defossez, A. 2020. “Social and Digital Resources: The Hindered Information Practices of Cancer Patients.” Health Research Practices in a Digital Context 4: 23–37. 10.1002/9781119779933.ch2.

[shil13863-bib-0011] Emirbayer, M. , and J. Goodwin . 1994. “Network Analysis, Culture, and the Problem of Agency.” American Journal of Sociology 99, no. 6: 1411–1454. 10.1086/230450.

[shil13863-bib-0012] Ezeh, A. , O. Oyebode , D. Satterthwaite , et al. 2017. “The History, Geography, and Sociology of Slums and the Health Problems of People Who Live in Slums.” Lancet 389, no. 10068: 547–558. 10.1016/S0140-6736(16)31650-6. 27760703

[shil13863-bib-0013] Fourchard, L. 2003. “Urban Slums Report: The Case of Ibadan, Nigeria.” In Understanding Slums: Case Studies for the Global Report on Human Settlements. https://nairametrics.com/wp‐content/uploads/2013/05/URBAN‐SLUMS‐REPORT‐IBADAN.pdf.

[shil13863-bib-0014] Freidson, E. 1988. Profession of Medicine: A Study of the Sociology of Applied Knowledge. Chicago, USA: University of Chicago Press.

[shil13863-bib-0015] Gamper, M. 2022. “Social Network Theories: An Overview.” In Social Networks and Health Inequalities, edited by A. Klärner , M. Gamper , S. Keim‐Klärner , I. Moor , and V. Vonneilich , 35–48. Cham, Switzerland: Springer International Publishing.

[shil13863-bib-0016] Garfinkel, H. 1964. “Studies of the Routine Grounds of Everyday Activities.” Social Problems 11, no. 3: 225–250. 10.2307/798722.

[shil13863-bib-0017] Garner, S. L. , T. Sudia , and S. Rachaprolu . 2018. “Smart Phone Accessibility and mHealth Use in a Limited Resource Setting.” International Journal of Nursing Practice 24, no. 1: e12609. 10.1111/ijn.12609. 29159919

[shil13863-bib-0018] Gillespie, B. , H. Allen , M. Pritchard , P. Soma‐Pillay , J. Balen , and D. Anumba . 2021. “Agency Under Constraint: Adolescent Accounts of Pregnancy and Motherhood in Informal Settlements in South Africa.” Global Public Health 17, no. 9: 1–14. 10.1080/17441692.2021.1981974. 34569422

[shil13863-bib-0019] Greif, M. J. , and F. N. A. Dodoo . 2015. “How Community Physical, Structural, and Social Stressors Relate to Mental Health in the Urban Slums of Accra, Ghana.” Health & Place 33: 57–66. 10.1016/j.healthplace.2015.02.002. 25754264

[shil13863-bib-0020] Harris, B. , M. Ajisola , R. M. Alam , et al. 2021. “Mobile Consulting as an Option for Delivering Healthcare Services in Low‐Resource Settings in Low‐and Middle‐Income Countries: A Mixed‐Methods Study.” Digital Health 7. 10.1177/20552076211033425. PMC8580492 34777849

[shil13863-bib-0021] Hiscock, J. 2013. Informal Interactions About Health: Connectedness, Surveillance and the Construction of a Moral Identity. United Kingdom: University of Manchester. ProQuest Dissertations Publishing. 10030806.

[shil13863-bib-0022] Horsfall, M. , M. Eikelenboom , S. Draisma , and J. H. Smit . 2021. “The Effect of Rapport on Data Quality in Face‐to‐Face Interviews: Beneficial or Detrimental?” International Journal of Environmental Research and Public Health 18, no. 20: 10858. 10.3390/ijerph18201085. 34682600 PMC8535677

[shil13863-bib-0023] Improving Health in Slums Collaborative . 2019. “A Protocol for a Multi‐Site, Spatially‐Referenced Household Survey in Slum Settings: Methods for Access, Sampling Frame Construction, Sampling, and Field Data Collection.” BMC Medical Research Methodology 19, no. 1: 109. 10.1186/s12874-019-0732-x. 31146676 PMC6543601

[shil13863-bib-0024] Javaid, M. , A. Haleem , and R. P. Singh . 2023. “ChatGPT for Healthcare Services: An Emerging Stage for an Innovative Perspective.” BenchCouncil Transactions on Benchmarks, Standards and Evaluations 3, no. 1: 100105. 10.1016/j.tbench.2023.100105.

[shil13863-bib-0025] Khalil, K. , P. Das , R. Kammowanee , et al. 2021. “Ethical Considerations of Phone‐Based Interviews From Three Studies of Covid‐19 Impact in Bihar, India.” Supplement, BMJ Global Health 6, no. S5: E005981. 10.1136/bmjgh-2021-005981. PMC8375446 34404691

[shil13863-bib-0026] Loignon, C. , T. Gottin , N. Rahem , et al. 2022. “Maternal Experience With Online Information on Parenting and Infant Care: Qualitative Findings From Quebec, Canada.” Journal of Child and Family Studies 31, no. 7: 1798–1808. 10.1007/s10826-021-02205-w.

[shil13863-bib-0027] Lubbers, M. J. , M. L. Small , and H. V. García . 2020. “Do Networks Help People to Manage Poverty? Perspectives From the Field.” Annals of the American Academy of Political and Social Science 689, no. 1: 7–25. 10.1177/0002716220923959.

[shil13863-bib-0028] Lythreatis, S. , S. K. Singh , and A. El‐Kassar . 2022. “The Digital Divide: A Review and Future Research Agenda.” Technological Forecasting and Social Change 175: 121359. 10.1016/j.techfore.2021.121359.

[shil13863-bib-0029] Morgan, D. L. 2012. “Focus Groups and Social Interaction.” In The Sage Handbook of Interview Research: The Complexity of the Craft, 2, edited by J. Gubrium , J. Holstein , A. Marvasti , and K. McKinney , 161–176. California: SAGE Publications, Inc.

[shil13863-bib-0030] Mpanje, D. , P. Gibbons , and R. Mcdermott . 2018. “Social Capital in Vulnerable Urban Settings: An Analytical Framework.” Journal of International Humanitarian Action 3, no. 1: 4. 10.1186/s41018-018-0032-9.

[shil13863-bib-0031] Mueller, J. , C. Jay , S. Harper , A. Davies , J. Vega , and C. Todd . 2017. “Web Use for Symptom Appraisal of Physical Health Conditions: A Systematic Review.” Journal of Medical Internet Research 19, no. 6: e202. 10.2196/jmir.6755. 28611017 PMC5487739

[shil13863-bib-0032] Mwaka, A. D. , F. M. Walter , S. Scott , J. Harries , H. Wabinga , and J. Moodley . 2021. “Symptom Appraisal, Help‐Seeking and Perceived Barriers to Healthcare Seeking in Uganda: An Exploratory Study Among Women With Potential Symptoms of Breast and Cervical Cancer.” BMJ Open 11, no. 2: e041365. 10.1136/bmjopen-2020-041365. PMC7925866 33550241

[shil13863-bib-0033] Nigeria Centre for Disease Control and Prevention . 2020. Infection Prevention and Control Reccomendations During Health Care Provision for Suspected and Confirmed Cases of Covid‐19. https://ncdc.gov.ng/ncdc.gov.ng/themes/common/docs/protocols/195_1599843797.pdf.

[shil13863-bib-0034] Nsor‐Anabiah, S. , M. U. Udunwa , and S. Malathi . 2019. “Review of the Prospects and Challenges of mHealth Implementation in Developing Countries.” International Journal of Applied Engineering Research 14, no. 12: 2897–2903.

[shil13863-bib-0035] Onuegbu, C. , J. Harlock , and F. Griffiths . 2023. “Use, Characteristics and Influence of Lay Consultation Networks on Treatment‐Seeking Decisions in Slums of Nigeria: A Cross‐Sectional Survey.” BMJ Open 13, no. 5: e065152. 10.1136/bmjopen-2022-065152. PMC10193090 37192804

[shil13863-bib-0036] Onuegbu, C. , M. Larweh , J. Harlock , and F. Griffiths . 2021. “Systematic Review of Lay Consultation in Symptoms and Illness Experiences in Informal Urban Settlements of Low‐Income and Middle‐Income Countries.” BMJ Open 11, no. 12: e050766. 10.1136/bmjopen-2021-050766. PMC8693092 34933858

[shil13863-bib-0037] Onwujekwe, O. , C. Mbachu , V. Onyebueke , et al. 2022. “Stakeholders’ Perspectives and Willingness to Institutionalize Linkages Between the Formal Health System and Informal Healthcare Providers in Urban Slums in Southeast, Nigeria.” BMC Health Services Research 22, no. 583: 1–14. 10.1186/s12913-022-08005-2. 35501741 PMC9059679

[shil13863-bib-0038] Perry, B. L. , and B. A. Pescosolido . 2015. “Social Network Activation: The Role of Health Discussion Partners in Recovery From Mental Illness.” Social Science & Medicine 125: 116–128. 10.1016/j.socscimed.2013.12.033. 24525260 PMC4110193

[shil13863-bib-0039] Pew Research Center . 2016. “Smartphone Ownership and Internet Usage Continues to Climb in Emerging Economies.” https://www.pewresearch.org/global/2016/02/22/smartphone‐ownership‐and‐internet‐usage‐continues‐to‐climb‐in‐emerging‐economies/.

[shil13863-bib-0040] Price, J. , M. Willcox , V. Dlamini , et al. 2021. “Care‐Seeking During Fatal Childhood Illness in Rural South Africa: A Qualitative Study.” BMJ Open 11, no. 4: E043652. 10.1136/bmjopen-2020-043652. PMC8094335 33926978

[shil13863-bib-0041] Queenan, J. , B. Gottlieb , D. Feldman‐Stewart , S. Hall , J. Irish , and P. Groome . 2018. “Symptom Appraisal, Help Seeking, and Lay Consultancy for Symptoms of Head and Neck Cancer.” Psycho‐Oncology 27, no. 1: 286–294. 10.1002/pon.4458. 28543939

[shil13863-bib-0042] Reeder, K. M. , J. L. Sims , P. M. Ercole , S. S. Shetty , and M. Wallendorf . 2017. “Lay Consultations in Heart Failure Symptom Evaluation.” SOJ Nursing & Health Care 3, no. 2: 1–7. 10.15226/2471-6529/3/2/00133. PMC5795614 29399657

[shil13863-bib-0043] Reñosa, M. D. C. , C. Mwamba , A. Meghani , et al. 2021. “Selfie Consents, Remote Rapport, and Zoom Debriefings: Collecting Qualitative Data Amid a Pandemic in Four Resource‐Constrained Settings.” BMJ Global Health 6, no. 1: E004193. 10.1136/bmjgh-2020-004193. PMC7798410 33419929

[shil13863-bib-0044] Rodrigues, C. F. 2020. “Self‐Medication With Antibiotics in Maputo, Mozambique: Practices, Rationales and Relationships.” Palmgrave Communications 6, no. 24: 1–12. 10.1057/s41599-020-0401-z.

[shil13863-bib-0045] Sarkar, A. 2020. “Everyday Practices of Poor Urban Women to Access Water: Lived Realities From a Nairobi Slum.” African Studies 79, no. 2: 212–231. 10.1080/00020184.2020.1781594.

[shil13863-bib-0046] Silver, L. , and C. Johnson . 2018. “Majorities in Sub‐Saharan Africa Own Mobile Phones, But Smartphone Adoption Is Modest.” Pew Research Centre: Global Attitudes & Trends. https://www.pewresearch.org/global/2018/10/09/majorities‐in‐sub‐saharan‐africa‐own‐mobile‐phones‐butsmartphone‐adoption‐is‐modest/.

[shil13863-bib-0047] Smith, D. , P. Newton , J. Berlin , and S. Barrett . 2020. “A Community Approach to Engaging Gypsy and Travellers’ in Cancer Services.” Health Promotion International 35, no. 5: 1094–1105. 10.1093/heapro/daz103. 31617902

[shil13863-bib-0048] Smith, K. P. , and N. A. Christakis . 2008. “Social Networks and Health.” Annual Review of Sociology 34, no. 1: 405–429. 10.1146/annurev.soc.34.040507.134601.

[shil13863-bib-0049] Sunikka‐Blank, M. , R. Bardhan , and A. N. Haque . 2019. “Gender, Domestic Energy and Design of Inclusive Low‐Income Habitats: A Case of Slum Rehabilitation Housing in Mumbai, India.” Energy Research & Social Science 49: 53–67. 10.1016/j.erss.2018.10.020.

[shil13863-bib-0050] Thomas, J. , and A. Harden . 2008. “Methods for the Thematic Synthesis of Qualitative Research in Systematic Reviews.” BMC Medical Research Methodology 8, no. 1: 45. 10.1186/1471-2288-8-45. 18616818 PMC2478656

[shil13863-bib-0051] United Nations Habitat . 2020. “World Cities Report 2020: The Value of Sustainable Urbanization.” https://unhabitat.org/world‐cities‐report‐2020‐the‐value‐of‐sustainable‐urbanization.

[shil13863-bib-0052] Van Der Heijden, J. , N. Gray , B. Stringer , et al. 2019. “Working to Stay Healthy’, Health‐Seeking Behaviour in Bangladesh’s Urban Slums: A Qualitative Study.” BMC Public Health 19, no. 1: 600. 10.1186/s12889-019-6750-0. 31101099 PMC6525448

[shil13863-bib-0053] Van De Vijver, S. , S. Oti , C. Oduor , et al. 2015. “Challenges of Health Programmes in Slums.” Lancet 386, no. 10008: 2114–2116. 10.1016/S0140-6736(15)00385-2. 26452707

[shil13863-bib-0054] Willan, S. , A. Gibbs , N. Shai , N. Ntini , I. Petersen , and R. Jewkes . 2020. “Did Young Women in South African Informal Settlements Display Increased Agency After Participating in the Stepping Stones and Creating Futures Intervention? A Qualitative Evaluation.” Social Science & Medicine 265: 113302. 10.1016/j.socscimed.2020.113302. 32890814

[shil13863-bib-0055] Zerbo, A. , R. C. Delgado , and P. A. González . 2020. “Vulnerability and Everyday Health Risks of Urban Informal Settlements in Sub‐Saharan Africa.” Global Health Journal 4, no. 2: 46–50. 10.1016/j.glohj.2020.04.003.

